# Sharp-Hook Acupuncture (*Feng Gou Zhen*) for Patients with Periarthritis of Shoulder: A Randomized Controlled Trial

**DOI:** 10.1155/2015/312309

**Published:** 2015-11-10

**Authors:** Laixi Ji, Haijun Wang, Yuxia Cao, Ping Yan, Xiaofei Jin, Peirui Nie, Chaojian Wang, Rangqian Li, Chunlong Zhang, Mingxiao Yang, Jie Yang

**Affiliations:** ^1^Shanxi College of Traditional Chinese Medicine, Jinzhong, Shanxi 030619, China; ^2^The Third Teaching Hospital of Shanxi College of Traditional Chinese Medicine, Jinzhong, Shanxi 030006, China; ^3^Chengdu University of Traditional Chinese Medicine, Chengdu, Sichuan 610072, China

## Abstract

The* Feng Gou Zhen* (sharp-hook acupuncture) as a traditional form of ancient acupuncture is said to be particularly effective for managing periarthritis of shoulder. We conducted this randomized controlled trial to evaluate the effectiveness of* Feng Gou Zhen* as an add-on compared to conventional analgesics for patients with PAS. 132 patients were randomly assigned in a 1 : 1 ratio to either a acupuncture group receiving sharp-hook acupuncture plus acupoint injection with conventional analgesics or a control group. Patients from both groups were evaluated at week 0 (baseline), week 1, and week 4. The primary outcome measure was the change from baseline shoulder pain, measured by Visual Analogue Scale at 7 days after treatment. Secondary outcome measures include the (i) function of shoulder joint and (ii) McGill pain questionnaire. The results showed that patients in acupuncture group had better pain relief and function recovery compared with control group (*P* < 0.05) at 1 week after treatment. Moreover, there were statistical differences between two groups in VAS and shoulder joint function and McGill pain questionnaire at 4 weeks after treatment (*P* < 0.05). Therefore, the sharp-hook acupuncture helps to relieve the pain and restore the shoulder function for patients with periarthritis of shoulder.

## 1. Background

Periarthritis of shoulder (PAS), or, namely, the frozen shoulder, is a common, disabling musculoskeletal disorder in middle-aged people. It commonly refers to a collection of pain symptoms in shoulders, plus motor function limitation, due to soft tissue abnormalities surrounding the affected shoulder [1]. It is mostly a aseptic inflammation of the musculoskeletal system. The PAS is prevailing in people of the age 40 to 60 years, and thus they were called “the forty's shoulder.” The population incidence of PAS is 2%–5% in the United States [2]. In China, the PAS in urban population accounts for 8% and three quarters are female patients [3]. Studies also demonstrated that inadequate treatment or inappropriate treatment led to severely reduced life quality and unsatisfactory work performance [4].

Nowadays, there are many treatment options for PAS, including intra-articular triamcinolone injection [5] and bupivacaine suprascapular nerve blocks [6]. These modern therapies are successfully employed in clinical practice for PAS treatment. However, the treatment effects are not always long-lasting and most of which disappear in 4 weeks after treatment approximately [7, 8]. Moreover, these treatment options may be associated with severe adverse events, such as infectious arthritis and cartilage damage, all of which may put the patient into certain risks of disease deterioration [9]. Therefore, to search for novel treatment modality is of great significance for the management of PAS.

In China, acupuncture is an effective traditional therapeutics that is originated in ancient China. During its over 3000 years of history of clinical practice, acupuncture treatment gets developed and evolved to treat various disorders. In recent decades, acupuncture as a complementary and alternative therapy has been increasingly recognized in western countries. Current evidence suggests that acupuncture is a valid intervention for treating pain [10, 11] and musculoskeletal disorders [12, 13]. There are also studies revealing that acupuncture triggers the endogenous opium and cannabinoids system to release several pain-ameliorating substances to reduce pain [14, 15]. Therefore, many PAS patients resorted to acupuncture therapy in China and other foreign countries. Though studies reported that acupuncture is effective for PAS, the strength of evidence and its recommendation is still weak. According to a recent Cochrane systematic review, the effect of acupuncture for shoulder pain and its function improvement is still inconclusive. Current little evidence is unable to affirm or refute the effectiveness of acupuncture for PAS. It is of great importance to conduct a clinical trial with rigorous methodology to assess acupuncture's effect.

**Figure 1:**
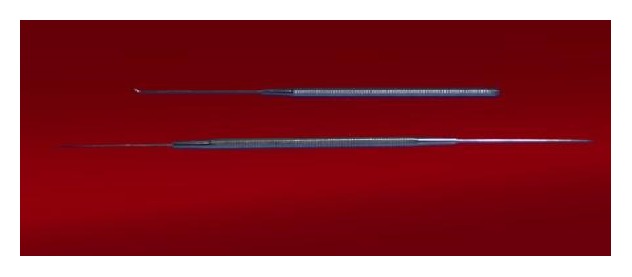
Illustration of the* Feng Gou Zhen* (sharp-hook needle).

Importantly, acupuncture is a complex of different needling therapies. Different acupuncture modalities or equipment may possess different treatment powers. The* Feng Gou Zhen* (sharp-hook needle) is a modified form of ancient nine needles as recorded in the Yellow Emperor's Internal Medicinal Cannon (as shown in Figure 1). The modification of this form of needle is initiated by Dr.* Huaitang Shi* who was a renowned acupuncturist in China, as well as the First Director of the Research Center of Acupuncture in Shanxi. The design of the* Feng Gou Zhen* is based on the three-edge sharp needle of ancient nine needles and the adding of certain features of the hook needle in folks. By integrating their characteristics, we invented the* Feng Gou Zhen* device. Our clinical observation indicates that the* Feng Gou Zhen* is very effective for treating lots of diseases, and PAS is just one of them. In our practice, we found that one session or two sessions of sharp-hook acupuncture treatment relieve the pain of PAS and recovers the function of shoulder joint. But, at present there is still no randomized controlled trial evaluating the effective of sharp-hook acupuncture for PAS. We therefore performed this one-center, randomized controlled, open-labeled trial to assess the effect of acupuncture as add-on for pain relief and joint function improvement in PAS.

## 2. Participants and Methods

### 2.1. Trial Design

This study is a one-center, randomized, controlled, and open-labeled trial that assesses the effect of sharp-hook acupuncture in addition to acupoint injection with conventional analgesics for treating PAS. Eligible patients were randomly assigned to either acupuncture group or control group in a 1 : 1 ratio.

### 2.2. Participants

All patients were recruited from the Anesthesiology Center and General Medicine Department of the* Shanxi* Acupuncture Research Center between October 2012 and October 2014. Shanxi Regional Ethics Review Committee on Traditional Chinese Medicine has approved all research procedures (2012LC-03) and has been registered in Chinese Clinical Trial Registry (ChiCTR-IOC-15006309). All included participants provided their written inform consent with signature. Patients were introduced to this trial by their doctors. The eligibility criteria for participants included the inclusion and exclusion criteria that were referred to in Sun et al.'s study [16] and listed as follows.

#### 2.2.1. Inclusion

Inclusion criteria include (1) shoulder pain for at least 1 month and less than 12-month duration; (2) appreciable restriction of both active and passive motions with abduction and flexion not exceeding 90° and external rotation not exceeding 30°; (3) pain at night, with inability to lie on the affected side; (4) age between 40 years and 65 years; (5) receiving no treatment in the last 4 weeks; (6) agreeing to cooperate with doctor's instructions of acupuncture; (7) providing written inform consent.

#### 2.2.2. Exclusion

Exclusion criteria include (1) history of major shoulder injury or surgery; (2) clinical or radiological evidence of other pathologies that could possibly account for symptoms; (3) patients with evidence of cervical radiculopathy, paresis, or other neurological changes in the upper limb on the involved side; (4) the presence of underlying fracture, associated inflammatory arthritis, known renal or hepatic disease, haematopoietic disorder, malignancy, any mental disorder likely to interfere with the course or assessment of the disease process; (5) painful arc between 40° and 120° abductions indicative of rotator cuff disease; (6) uncontrolled diagnosed neurological diseases, immunodeficiency, bleeding disorders, and allergies; (7) uncontrolled medical conditions which are unfit for acupuncture; (8) patient receiving acupuncture currently or received acupuncture 2 weeks prior to enrollment; (9) women in lactation, pregnant women, or with plans to get pregnant in the coming half year; (10) patients taking drugs such as NSAIDs or other pain killers that can affect the outcomes; (11) patients undergoing other trials.

The included patients were treated in the outpatient clinics of these departments. An individual researcher collected the data in clinics.

### 2.3. Interventions

Patients in acupuncture group received sharp-hook acupuncture plus acupoint injection with conventional analgesics. For control group, there was only acupoint injection with conventional analgesics. There was only one treatment session in both groups. All treatments were performed by acupuncturists with over 10 years of clinical experience.

#### 2.3.1. Acupuncture Group

The procedures of sharp-hook acupuncture treatment were listed as follows.

1. A collection of 5 acupoints, including A-Shi point,* Jianyu* (LI15),* Jianliao* (SJ14),* Jianzhen* (SI9), and* Jianqian* (Ex-UE), was selected and then marked by methylrosanilinium chloride when the patient was in a sitting position. The locations of acupoints were referred to in the Chinese Standard of acupoint, the GB/T 30233-2013. The local skin of the selected acupoints was sterilized by 75% ethanol.
2. Then, we prepared the conventional analgesics for acupoint injection to block nerve by mixing 2% lidocaine of 3 mL (*Zhuo Feng* pharmaceutics, Zhengzhou, China), vitamin B_12_ of 0.5 mg (*Tian Jin* Pharmaceutics, Tianjin, China), triamcinolone acetonide acetate of 20 mg (*Xian Ju* Pharmaceutics, Xianju, Zhejiang), and 0.9% saline of 3 mL (the 4th Pharmaceutics of* Shi Jia Zhuang*, Shijiazhuang, Hebei). After that, every marked acupoint was injected 1 mL of the drug mixture.
3. We then inserted the sharp-hook needle to the subcutaneous level of these acupoints. The depth of insertion was depending on the depth and severity of affected area.
4. The needle was kept a 45°–75° angle to the surface of the skin and was constantly pulled in and out for 3–5 times to stretch the fibers within the affected tissue, but with the needle retained in skin. For extreme tenses and nodules, it was necessary for the acupuncturist to intersect the fascia, muscle fibers, and ligament surrounding the rigid tissue, till the tension was removed.
5. The needle was returned back to the angle and position as it was inserted and then was quickly withdrawn. After that, we applied a cup over the cut to let the blood stasis out. At last, the cut was disinfected and we applied a piece of BAND-AID.

To be noted, “A-Shi point” refers to the most representative point of PAS symptoms, mostly pain, on certain body surface, where it hurts the most when pressed on with finger(s)/treatment equipment. Therefore, no fixed location of A-Shi point can be defined. Mostly, its identification is depending on the location of disease, the place where the organ was affected and where tissue was harmed.

#### 2.3.2. Control Group

The procedures of treatment were the same as (1) and (2) steps in acupuncture group. The acupoints and analgesics used in control group were identical to acupuncture group.

### 2.4. Outcomes

The primary outcome was the pain relief of shoulder as measured by Visual Analogue Scale at 1 week after treatment. The secondary outcomes included (1) the function of shoulder joint as measured by the physical examination score for shoulder and (2) McGill pain questionnaire. All outcome measurements were assessed at 0 week, 1 week, and 4 weeks and were collected by an individual researcher in clinics or by telephone interview. The safety issue of sharp-hook acupuncture for patients with periarthritis of shoulder was assessed by any related adverse events.

### 2.5. Sample Size

Sample size was calculated by a G^*∗*^Power 3 software (Institute for Experimental Psychology, Heinrich-Heine-University, Germany). For this trial, it has been determined prospectively that *α* = 0.05 and 1 − *β* = 0.90. According to a previous trial on acupuncture for PAS [17], a total of 120 participants will be included in this trial for a compensation to 15% dropout rate, with 60 patients in each group.

### 2.6. Randomisation

In this trial, participants were randomly assigned to either acupuncture group or control group in a 1 : 1 ratio. Random number sequence was computationally generated by clinical researcher using the SPSS 16.0 software. In the random number sequence, number “1” represents acupuncture group, while number “2” stands for the control group. Each random number was carefully concealed by the clinical researcher in an sequentially numbered opaque envelope which was not permitted for acupuncturist to unfold until eligible patients were included in this trial with written informed consent. After a patient was enrolled in the trial by the clinical trial communicator, the clinician would open one of the opaque envelopes according to his/her inclusion order and then further assign the patient to either acupuncture or control group.

### 2.7. Blinding

As an open-labeled clinical trial, the patients and clinicians would not be blinded in the whole treatment process. Patients in each group knew which treatment approach they would receive; they were required to cooperate with their physicians or therapists prior to treatment. The assessment of clinical effectiveness was performed through telephone interview by a clinical assessor who was masked to the treatment assignment. While in data collection and analysis stage, the clinical researcher, assessor, and statistician were, respectively, separated from each other.

### 2.8. Statistical Methods

All main analyses (with SPSS 16.0) were based on the intention-to-treat population which automatically imputed the missing data according to the last visit data. Additionally, a per protocol analysis was done including only patients with no major protocol violations by the end of 4 weeks after randomization. The demographic, clinical, and outcome variables at baseline were described by using means and standard deviations for continuous variables. Two-sample *t*-test analysis of variance was used to compare baseline data and difference between groups. Paired *t*-test analyses were used to compare the pre- and posttreatment data within group. A two tailed *P* < 0.05 was considered significantly different.

## 3. Results

**Figure 2:**
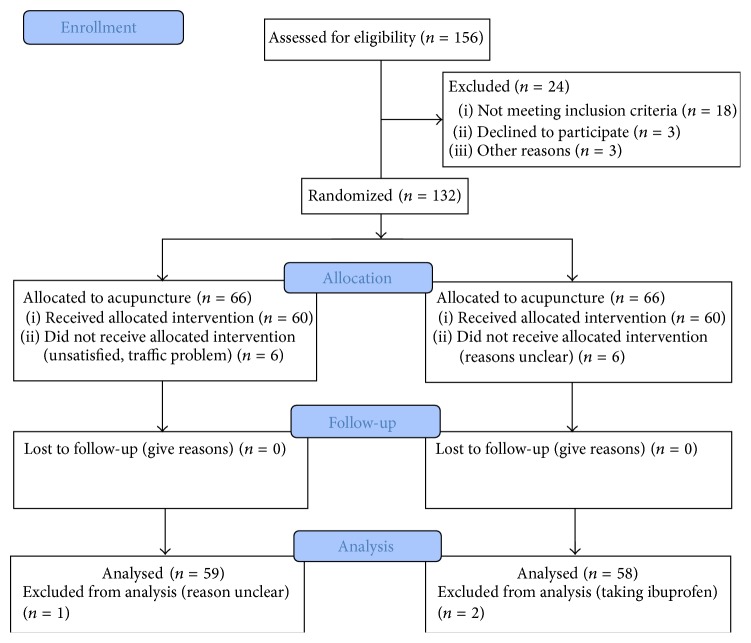
Trial flow chart.

156 patients were enrolled in this study, but only 132 eligible ones were included to received treatment. 24 patients were excluded due to various reasons. For the included patients, 120 completed the trial (see Figure 2). Six patients in acupuncture group discontinued the study due to unsatisfaction, traffic problem; 6 patients in control group abandoned the treatment due to reasons that are unclear. Due to the very short follow-up period, all patients returned follow-up data. In data analysis, 59 patients in acupuncture group and 58 patients in control group were included in ITT analysis.

### 3.1. Baseline Data

The demographic information and baseline comparison between groups were shown in Table 1. There was no significant difference in age, gender, and disease history between groups (*P* > 0.05). Moreover, the pain intensity no matter measured by VAS or McGill pain questionnaire and the shoulder joint function score measured by general physical examination were not significant between acupuncture group and control group (*P* > 0.05).

### 3.2. Outcomes and Estimation

The pain intensity and joint function score at 1 week and 4 weeks after randomization were listed in Table 2. The result showed that both acupuncture and conventional analgesics significantly reduced pain intensity of PAS measured by VAS as compared with their respective baseline condition (*P* < 0.05). The comparison between two groups showed that, at 1 week after randomization, the pain intensity measured by VAS in acupuncture groups showed more significant reduction than control group (*P* < 0.0001). Pain intensity measured by McGill pain questionnaire showed similar significant reduction in both groups (*P* < 0.05). At 1 week after randomization, acupuncture significantly reduced pain intensity measured by McGill pain questionnaire compared with control group (*P* < 0.01). Moreover, the function of shoulder joint was significantly improvement in both groups as compared with baseline (*P* < 0.05). The comparison between acupuncture group and control group showed that shoulder joint function was significantly improvement in sharp-hook acupuncture group as compared with control group at 1 week after randomization.

**Figure 3:**
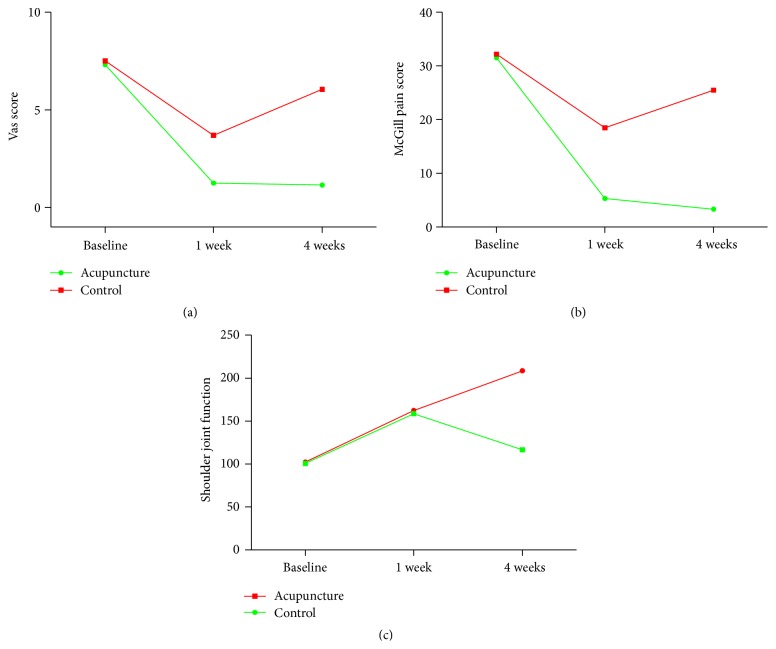
Changes in pain intensity and shoulder joint function in trial process and follow-ups.

However, in the 4-week follow-up, the pain intensity and shoulder joint function of patients in control group relapsed but still with statistical difference as compared with baseline. As to acupuncture group, the pain intensity and shoulder joint function were significantly improved as compared with baseline and 1 week follow-up (*P* < 0.05), suggesting that the treatment effect lasted from the 1 week to the 4 weeks after randomization. Furthermore, there were more significant reductions in pain intensity and shoulder joint function for acupuncture group at 4 weeks after randomization, as compared with control group (*P* < 0.05). The changes in pain intensity as measured by VAS or McGill pain questionnaire and the shoulder joint function score were shown in Figure 3. No adverse events occurred in the trial process or during the 4-week follow-up. Therefore, sharp-hook needle was deemed to be a safe treatment for these patients included in this trial.

## 4. Discussions

The periarthritis of shoulder is a prevailing musculoskeletal disorder which commonly pathologically encompasses aseptic inflammation and soft tissue adherence in shoulder joint. Though not a fetal disorder or major disease like cancer, angina pectoris, stroke, and so forth, the PAS seriously causes pain, leads to notable motor function limitation of the affected joint, and subsequently endangers people's life quality. Therefore, the treatment of PAS is of great significance to increase people's life quality and advance public health. However, conventional treatment of modern medicine cannot withdraw the symptoms and harmfulness of PAS completely and satisfactory, most of which are associated with adverse events and side effect. It is therefore of great importance to introduce some other therapeutics as complementary and alternatives to help boost the treatment effect and increase patient's compliance.

Acupuncture in China has been applied in PAS treatment for a long history. Now with culture communication and immigration, people in western world started to use acupuncture treating PAS in recent decades. However, the evidence base of the effectiveness of acupuncture for PAS is not firm. A recent systematic review suggested that the reasons for inclusive statements were due to the inadequacy in trial design and implementation [18]. Therefore, in this study, we used a prospectively defined trial protocol to evaluate the effect of a form of acupuncture for PAS. Due to the significant features of sharp-hook acupuncture, we were unable to perform certain blinding measures as suggested by standards of the Good Clinical Practice. We therefore employed the open-labeled design regardless of the placebo effect caused by unblinding. In the whole process, clinical practitioners, trial researchers, data collectors, and statisticians were independent of each other. The clinical practitioners and trial researchers had no access to clinical database and therefore the clinical data was unavailable to them. Moreover, the allocation information was completely masked for the assessors and statisticians. This step eliminates bias caused by researcher's preference and therefore ensures the internal validity of the results. On the other hand, this study demonstrated the application of an explanatory trial to assess the efficacy of the sharp-hook acupuncture for PAS. The patients were selected according to very rigorous criterion, which may increase the internal validity but decrease the external validity to some extent. The treatment form in experimental group and the acupuncture group included both acupuncture and conventional therapy, which is a mimic of the real work practice situation. Therefore, this design to some extent increased the external validity of the study. Future pragmatic trials in a real world environment are warranted for they can add more valuable findings.

This randomized controlled trial demonstrated that sharp-hook acupuncture therapy as an add-on boosted the analgesic effect of conventional nerve block and significantly increased patients' shoulder joint function. The difference in treatment effect observed in 4 weeks between acupuncture and control group may reflect the strength and importance of sharp-hook acupuncture. The result of this study is consistent with a previous study reporting that acupuncture plus shoulder exercise experienced significant improvement after treatment compared with exercise only [16]. In their study, they observed that the effect of acupuncture added to exercise sustained from 6 weeks after randomization to 20 weeks, suggesting a long-term effect of acupuncture. In our study, the focus is on the instant analgesic effect. We found that sharp-hook acupuncture led to significant reduction in pain and improvement in shoulder function. The 4-week follow-up indicates that the effect of sharp-hook acupuncture increases as the time passes. This response is consistent with the aforementioned study about the relative persistent effect of acupuncture. In addition, several other studies and reviews also confirmed our result [19–22].

Moreover, the superiority of sharp-hook acupuncture over nerve block by acupoint injection with conventional analgesics is not odd. The PAS is a disease not only with aseptic inflammation but also with severe soft tissue deformations [23–25]. Imaging studies suggested that there were significant pathological deformations in the local area of the affected shoulder of PAS patient [26, 27]. These pathological manifestations include capsulitis of the shoulder, trigger point [28] or pressing pain point, tensions, rigid muscle, or even nodes. In traditional Chinese medicine, this pathological presence was regarded as a convergence of unsmooth Qi and blood, blood stasis, and hot or cold phlegm. These pathological factors blocked the meridians and channels to cause pain and limited joint function. In modern medicine, the treatment emphasizes mostly emphasized local nerve block to stop pain with ignorance of the pathological presences, which may contribute to the relapse of pain and function limitation as observed in control group. While this trial applied three acupoints of the three Yang meridians of hand (namely, three acupoints for shoulders), accompanied with an extra-point Jianqian and the local A-Shi point to dredge the meridian, activate Qi and blood to remove pain. Moreover, the sharp-hook acupuncture needle is very unique in shape which enables it to be very convenient to remove or intersect the soft tissue adherence. In clinical practice, the sharp-hook acupuncture needle was used to deprive the functional soft tissue from adhering tissue, then to dissect the pathological scar within the affect joint and intersect the connections of the pathological nodes, according to blunt dissection and sharp dissection. The location of soft tissue and function of shoulder joint were restored by this procedure [29]. It is speculated that the dissection of these pathological presences by sharp-hook acupuncture needles is essential to the sustainable effect of sharp-hook acupuncture observed in the acupuncture group as compared with control group.

Safety is a very important issue for no matter novel therapy or traditional treatments. Actually, we have assessed the safety of sharp-hook needle. It is assessed by the adverse events reported by patients and doctors. We have a specific section in our case report form called “adverse events,” in which any adverse events including bleeding, hematoma, dizziness, faint, and nausea would be recorded if developed. This helps us to address the safety issue. However, there is no adverse events reported in our patients. It is true that the three-edge hook is thick and long in some cases. But the fact is that all our treatments were performed on the basis of local anesthesia by acupoint injection of 2% lidocaine. In addition, all practitioners are very experienced clinical doctors. Their clinical skills and communication ability are critical in this study. These are the reasons why the patients safety was guaranteed and their rights were respected. Thus, the sharp-hook needle is safe for PAS patients when handled with appropriateness.

Besides, the sharp-hook acupuncture needle invented by our team can also be used to perform blood-letting therapy by puncturing meridians. It is made of stainless metal, with a 14 cm needle body. The tip and the body of the needle forms a 110° angle. The needle tip is a three-edge hook with a length of 3 mm approximately, which makes it very subtle and easy for blood-letting and adherence dissection. It overcomes the limitations in superficial insertion and thus can be used to solve problems in deep tissue [29–32]. In this trial, acupuncture group also involved blood-letting and therefore may regulate blood circulation and the levels of proinflammatory, inflammatory, and anti-inflammatory factors to further improve PAS symptoms. For these reasons mentioned above, we therefore can rule out the possibility that the difference between groups was solely generated by the placebo effect.

Taken together, this study demonstrated that the* Feng Gou Zhen*, the sharp-hook acupuncture needle helps us to relieve pain and restore shoulder function for patients with periarthritis of shoulder. The present trial added good evidence to support the use of the* Feng Gou Zhen* as add-on to conventional nerve block for the treatment of periarthritis of shoulder.

## Figures and Tables

**Table 1:** Baseline information of two groups (mean ± SD).

	Acupuncture group	Control group	*P* value
Age (years)	51.2 ± 7.1	49.6 ± 9.7	0.3102
Gender (male/female)	26/33	28/30	0.6517
Disease history (month)	15.2 ± 8.3	14.9 ± 9.5	0.8559
Pain intensity measured by VAS	7.32 ± 1.51	7.51 ± 1.43	0.4862
McGill pain questionnaire	31.52 ± 4.53	32.21 ± 4.15	0.3923
Shoulder joint function score	102.42 ± 4.88	100.68 ± 5.52	0.0733

**Table 2:** Pain relief and shoulder joint function recovery at 1 week and 4 weeks after randomization.

	Acupuncture group	Control group	*P* value
1 week after randomization			
Pain intensity measured by VAS	1.25 ± 0.36	3.695 ± 1.16	<0.0001
McGill pain questionnaire	5.32 ± 1.24	18.48 ± 2.65	<0.0001
Shoulder joint function score	162.45 ± 10.65	158.68 ± 8.56	0.0372
4 weeks after randomization			
Pain intensity measured by VAS	1.15 ± 0.30	6.05 ± 1.31	<0.0001
McGill pain questionnaire	3.32 ± 1.18	25.48 ± 3.92	<0.0001
Shoulder joint function score	208.65 ± 12.95	116.52 ± 9.86	<0.0001
